# Macrophage dysregulation in medication-related osteonecrosis of the jaw: jaw-specific osteoimmune mechanisms and therapeutic perspectives

**DOI:** 10.3389/fimmu.2026.1884353

**Published:** 2026-10-20

**Authors:** Quan Wang, Shensui Li, Changfeng Linghu, Weijun Zhou, Zhenglong Tang

**Affiliations:** 1 College of Stomatology of Guizhou Medical University, Guiyang, China; 2 Key Laboratory of Oral Disease Research, Guizhou Medical University, Guiyang, China; 3 Department of Oral and Maxillofacial Surgery, Stomatological Hospital of Guizhou Medical University, Guiyang, China

**Keywords:** bone remodeling, efferocytosis, inflammation, macrophages, medication-related osteonecrosis of the jaw, osteoimmunology, protein prenylation

## Abstract

Medication-related osteonecrosis of the jaw (MRONJ) is a refractory complication of antiresorptive and antiangiogenic therapies. Its jaw-specific susceptibility and persistent inflammatory features are not fully explained by traditional hypotheses centered on suppressed bone remodeling or impaired angiogenesis. From an osteoimmunological perspective, this review proposes that MRONJ may be more accurately understood as an immune-mediated failure of healing within a jaw-specific osteoimmune niche, in which pathological macrophage reprogramming arises from the interplay between pharmacological exposure and the local microenvironment. Mechanistically, nitrogen-containing bisphosphonates inhibit the mevalonate pathway and disrupt the isoprenylation of small GTPases, including Rac1 and RhoA, resulting in cytoskeletal dysfunction that may impair macrophage efferocytosis. Consequently, persistent necrotic and apoptotic debris may provide ongoing danger signals that sustain NLRP3 inflammasome activation in susceptible lesions, thereby disrupting reparative coupling among bone, vasculature, and mucosa. In parallel, the unique jaw microenvironment, characterized by high local drug accumulation, biofilm colonization, and hypoxia, creates a high-burden inflammatory niche that further impairs the macrophage clearance–resolution program. Finally, this review highlights potential translational strategies, including metabolic rescue, specialized pro-resolving mediators (SPMs), and targeted local delivery of immunomodulatory biomaterials. Together, these approaches support a therapeutic roadmap centered on inflammation resolution and mucosal sealing as key clinical endpoints.

## 1 Introduction

Medication-related osteonecrosis of the jaw (MRONJ) is a refractory complication of antiresorptive and antiangiogenic therapies that develops specifically in the jawbones (1). Its incidence is below 0.1% in patients with osteoporosis but may reach 15% in patients with advanced malignancies receiving high-dose antiresorptive or related oncologic therapies (2, 3). With the expanding use of anticancer targeted agents and immunotherapies, the population at risk continues to grow, posing increasing challenges to clinical management (4). However, traditional hypotheses centered on “oversuppression of bone remodeling” or “impaired angiogenesis” do not fully resolve two fundamental questions: why does systemic drug exposure result in such striking jaw-specific susceptibility, and why are lesions so often characterized by persistent inflammation and complex biofilm-associated infection rather than simple aseptic necrosis (5–7)?

To overcome this conceptual impasse, the field must move beyond a narrow focus on bone metabolism and adopt the broader framework of osteoimmunology (8, 9). The jawbones are characterized by exceptionally high bone turnover and continuous exposure to the oral microbiota, making local homeostasis particularly dependent on effective immune surveillance and regulation. During physiological healing, macrophages orchestrate the transition from inflammation to repair by clearing damaged cells through phagocytosis and efferocytosis and releasing pro-resolving mediators that promote tissue restoration (10, 11). However, the ischemic, hypoxic, and metabolically altered local environment of the jaw can readily shift the activation threshold and functional state of myeloid cells, thereby disrupting the normal resolution program (12). In this context, MRONJ may be more usefully viewed not only as a metabolic bone disorder but also as an immune-mediated failure of healing (13, 14). Although previous studies have recognized the contribution of inflammation, it has often been considered a secondary consequence of osteonecrosis. Existing reviews have largely focused on osteoclast suppression, impaired angiogenesis, infection, and mucosal injury, whereas the role of macrophages has often been discussed mainly within a simplified polarization framework. The dynamic linkage among efferocytosis, inflammation resolution, and reparative coupling has not yet been fully integrated into a unifying model of MRONJ pathogenesis (15). A deeper understanding of how MRONJ-associated agents induce pathological macrophage reprogramming, particularly across the linked processes of efferocytosis, inflammation resolution, and reparative coupling, may therefore be critical to resolving this longstanding puzzle.

In this review, we first summarize the jaw-specific osteoimmune microenvironment that underlies susceptibility to MRONJ. We then discuss how MRONJ-associated drugs reprogram macrophage inflammatory and clearance functions, particularly by impairing efferocytosis, and thereby contribute to failed reparative coupling. Finally, we examine emerging translational strategies targeting macrophage reprogramming, with particular emphasis on specialized pro-resolving mediators (SPMs) and immunomodulatory biomaterials as potential approaches to restore immune homeostasis and overcome current therapeutic limitations in MRONJ.

## 2 Potential mechanisms underlying the jaw-specific predilection of MRONJ: a unique osteoimmune microenvironment

The jaw-specific predilection of MRONJ does not arise from a single determinant, but rather from the convergence of multiple factors, including the unique developmental origin and high metabolic turnover of the jawbones that favor local drug accumulation (9, 16), the functional specialization of bone-resident macrophages (17), and a local microenvironment shaped by oral microbial exposure together with hypoxic and acidic stress. In this section, we examine the osteoimmunological basis of jaw susceptibility from three interrelated dimensions: anatomy, metabolism, and immunity.

### 2.1 Anatomical features and high metabolic turnover of the jaw: preferential drug deposition

The distinctive embryonic origin of the jawbones confers unique cellular phenotypes and physiological properties. To withstand continuous masticatory stress and periodontal functional loading, the alveolar bone must maintain exceptionally high bone turnover and frequent osteoblast–osteoclast coupling (18). Because nitrogen-containing bisphosphonates and related agents exhibit high affinity for hydroxyapatite in the bone matrix, this high-turnover state promotes preferential deposition and accumulation of these drugs in the jawbones (19).

Against this anatomical and metabolic backdrop, local trauma, such as tooth extraction, often acts as an initiating event in MRONJ. When trauma triggers acute local bone resorption, matrix-bound drugs are abruptly released, generating a high-concentration toxic microenvironment at the wound site. Macrophages, including resident OsteoMacs and recruited monocyte-derived populations, are among the earliest immune cells involved in wound debridement and may engulf drug-laden bone debris, thereby sustaining bystander injury. This trauma-induced burst release of matrix-stored drugs may not only promote early macrophage apoptosis but also compromise phagocytic and clearance functions, thereby laying the foundation for impaired inflammation resolution and failed reparative coupling (20).

### 2.2 Macrophage heterogeneity: depletion of resident populations

Macrophage composition within the jaw microenvironment is dynamically altered, with depletion of resident populations and pathological accumulation of recruited macrophages (21). Bone-resident macrophages, also known as OsteoMacs, adhere to the bone surface and form canopy-like structures over osteoblasts, where they contribute to bone homeostasis by clearing osteogenic debris and secreting osteoinductive factors (17, 22).

OsteoMacs may be particularly vulnerable to local drug-rich conditions because of their intimate association with bone surfaces, potentially contributing to early disruption of osteoblast-supportive signals. Their loss may disrupt osteoblast-associated coupling signals and contribute to delayed extraction socket healing (23). At the same time, the resident macrophage compartment becomes progressively depleted (24). Under the combined pressure of drug exposure and local inflammatory stimuli, recruited macrophages are more likely to maintain a pro-inflammatory state, whereas their transition toward reparative programs is impaired. This shift establishes a self-perpetuating cycle of unresolved inflammation and failed tissue repair (25).

### 2.3 Local microenvironmental factors: microbial colonization, hypoxia, and acidosis

Beyond cellular composition, the distinctive anatomical structure and physicochemical properties of the jaw create conditions that may facilitate disease progression. Periodontal tissue represents a relatively vulnerable interface in the oral barrier, and disruption caused by tooth extraction or periodontitis can permit oral microbiota to invade the marrow space and form biofilms on necrotic bone surfaces (16). Bacterial components, particularly lipopolysaccharide, can persistently activate TLR4 signaling, thereby sustaining inflammatory responses and interfering with immune regulation and the initiation of resolution (7).

More importantly, these biological stimuli may act synergistically with local physicochemical stress to promote disease progression. Tissue hypoxia caused by microvascular injury and inflammatory edema can activate HIF-1α signaling in macrophages, favoring a metabolic shift toward glycolysis and thereby reinforcing a pro-inflammatory state (26). In addition, local acidosis induced by bacterial metabolites and excessive glycolysis may impair macrophage phagocytic receptor function and cytoskeletal dynamics (27). Within such a hostile microenvironment, pro-resolving signaling through the FPR2–RhoA axis may become functionally constrained, potentially limiting the cytoskeletal rearrangement required for efficient efferocytosis even in the presence of mediators such as RvD1 (14, 28). The resulting decline in efferocytic capacity may interrupt the clearance–resolution program and contribute to the refractory phenotype of MRONJ. Clinically, MRONJ is reported more often in the mandible (approximately 75%) than in the maxilla (approximately 25%), with both jaws involved in approximately 4.5% of cases (1). These data show a mandibular predilection, although MRONJ can affect either jaw. Site-related differences in vascularity, oxygenation, local drug exposure, and immune burden may therefore shape, but not determine, macrophage fate and healing (**Figure 1**).

**Figure 1:**
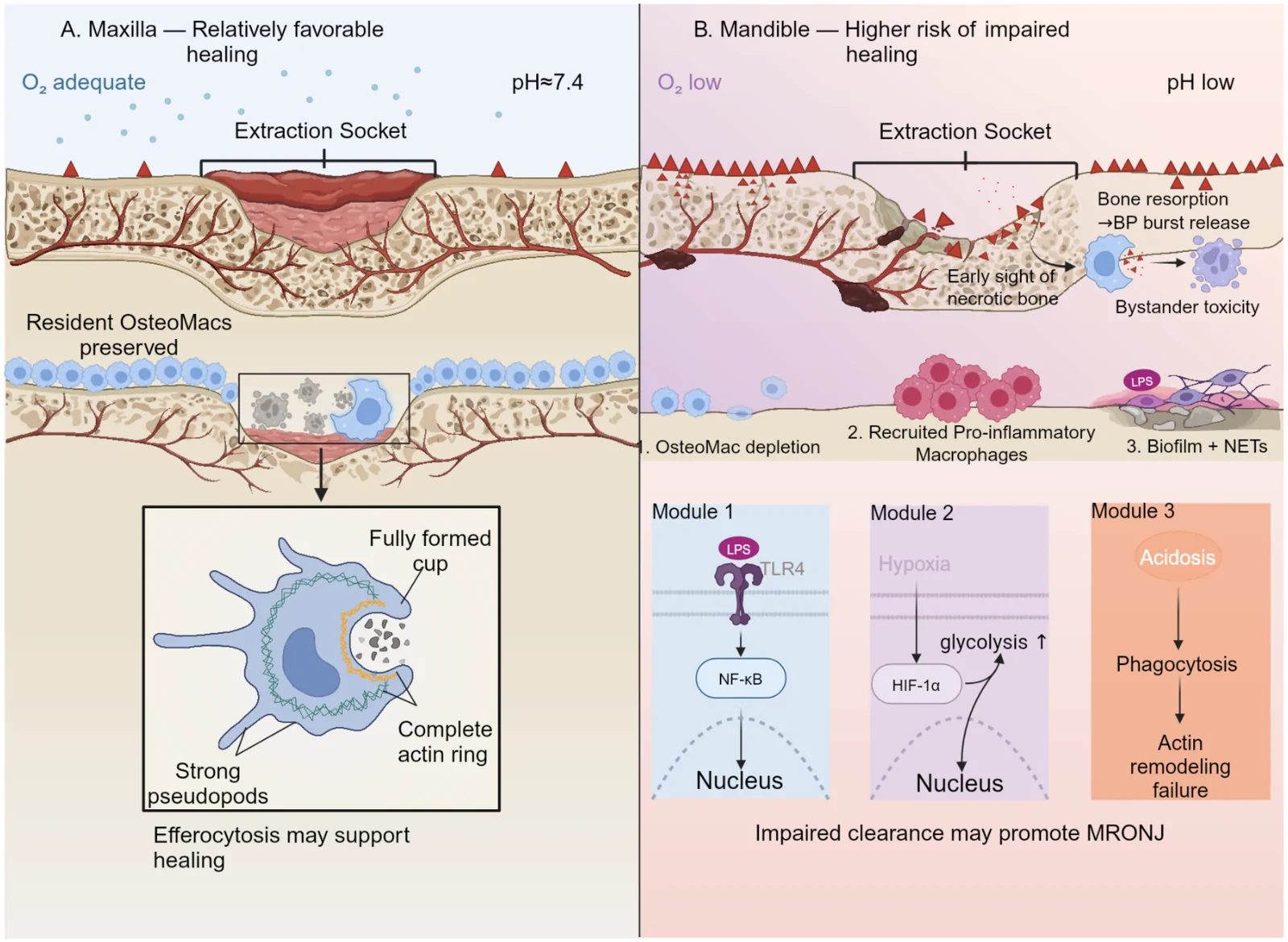
Proposed differences in post-extraction microenvironments and macrophage responses between the maxilla and mandible under bisphosphonate therapy. **(A)** More favorable vascularity and oxygenation in the maxilla may preserve OsteoMacs and support efferocytosis and tissue repair (11, 16, 17, 24, 28). **(B)** In the mandible, bisphosphonate release during bone resorption, hypoxia, acidosis, and biofilm-derived signals may promote OsteoMac depletion, the accumulation of pro-inflammatory macrophages, and defective clearance (7, 20, 26–30). These panels show differences in susceptibility rather than fixed healing outcomes in either jaw. Created in BioRender. Quad, W. (2026) https://BioRender.com/q89gxek.

## 3 Drug classes as immune-reprogramming factors: how MRONJ-associated agents “rewrite” macrophage function

MRONJ-associated drugs not only suppress bone remodeling, but also act as potent immune-reprogramming factors that profoundly reshape macrophage metabolic networks, cytoskeletal dynamics, and the temporal program of resolution at the very early stages of tissue injury (1). Given the uniquely high microbial burden of the jaw microenvironment, even subtle deviations in the macrophage clearance–resolution–repair program (10) can be readily amplified into persistent inflammation and uncoupled tissue repair (8). In this section, we systematically examine how different classes of MRONJ-related drugs precisely reprogram core macrophage functional modules at the molecular level, as well as how these effects are pathogenically amplified by local stress signals.

### 3.1 A central driver: bisphosphonate-induced mevalonate pathway blockade and dual paralysis of cytoskeletal and metabolic programs

The macrophage toxicity of nitrogen-containing bisphosphonates (N-BPs), such as zoledronic acid, does not primarily arise from direct cytotoxicity, but instead stems from the precise blockade of a central lipid metabolic hub, namely the mevalonate pathway (19). By specifically inhibiting farnesyl pyrophosphate synthase (FPPS), N-BPs markedly reduce the biosynthesis of key isoprenoid intermediates, including farnesyl pyrophosphate (FPP) and geranylgeranyl pyrophosphate (GGPP) (31). This metabolic interruption triggers a critical molecular cascade: small GTPases such as Rac1 and RhoA fail to undergo prenylation because of an insufficient GGPP supply. Without their hydrophobic lipid tails, these key cytoskeletal regulators are unable to anchor to the inner leaflet of the plasma membrane, resulting in spatial uncoupling of signal transduction at the membrane interface (32). This mislocalization directly leads to profound impairment of actin cytoskeletal dynamics in macrophages.

At a deeper level, this defect also disrupts pro-resolving signaling. Even when specialized pro-resolving mediators are present locally, such as when Resolvin D1 engages its cognate receptor FPR2 to initiate the resolution program, signal propagation may be aborted because its critical downstream effector, RhoA, remains unprenylated, cytosolic, and functionally inactive. As a result, macrophages fail to undergo the cytoskeletal rearrangement required for phagocytic cup formation, and local clearance of necrotic bone fragments and apoptotic cells is markedly compromised, thereby prolonging the persistence of damaged tissue at the lesion site (28).

From the perspective of immunometabolism, this mechanism is even more far-reaching. Mevalonate pathway blockade not only compromises cytoskeletal architecture, but also drives metabolic reprogramming (27). In a chronically inflamed microenvironment, impairment of this pathway acts in concert with pathological stress to shift macrophage energy production away from efficient mitochondrial oxidative phosphorylation (OXPHOS) toward the less efficient yet pro-inflammatory glycolytic pathway (33). This metabolic switch has dual consequences. First, glycolysis alone generates insufficient ATP to sustain the energetically demanding process of continuous efferocytosis. Second, disruption of the tricarboxylic acid cycle leads to pathological accumulation of metabolites such as succinate. Succinate can inhibit prolyl hydroxylase activity and thereby stabilize hypoxia-inducible factor-1α (HIF-1α) even under normoxic conditions, a mechanism that may promote transcription of downstream pro-inflammatory genes such as IL-1β in MRONJ-associated inflammatory settings. Through this metabolic remodeling, macrophages become functionally locked into a high-demand yet low-efficiency pro-inflammatory state (34, 35).

Notably, this complex pathological phenotype may be partially rescued by exogenous supplementation with geranylgeraniol (GGOH). As a membrane-permeable lipid precursor, GGOH bypasses the FPPS blockade and restores the supply of isoprenoid substrates required for the prenylation of downstream small GTPases, thereby partially rescuing macrophage morphology and phagocytic function (28, 36). This “metabolic rescue” experiment provides mechanistic support for a contributory role of mevalonate pathway blockade in nitrogen-containing bisphosphonate-induced macrophage dysfunction.

### 3.2 Synergistic amplifiers: denosumab and antiangiogenic agents induce phase mismatch and hypoxic stress

Beyond the direct intracellular toxicity of bisphosphonates, RANKL inhibitors (such as denosumab), antiangiogenic agents, and glucocorticoids can impose multiple injurious pressures on macrophages by reshaping the local microenvironment, thereby generating potent synergistic pathogenic effects.

First, while specifically neutralizing RANKL, denosumab may also alter macrophage-relevant osteoimmune signaling, although its direct effects on macrophage programming in MRONJ remain less clearly defined than those of nitrogen-containing bisphosphonates (37, 38). Sustained blockade of this signaling axis not only disrupts the physiological coupling between bone resorption and bone formation (39), but also deprives macrophages of the remodeling-related cues required during the reparative phase. This phase mismatch disrupts the reconstruction cues needed for the transition from debridement to resolution, forcing the wound to remain trapped in a state of chronic stress (40, 41).

Second, the addition of antiangiogenic agents or multitarget tyrosine kinase inhibitors (TKIs) may contribute to a more persistent hypoxic microenvironment within the lesion (42). This severe stress strongly activates the hypoxia–metabolism–immunity axis, exemplified by HIF-1α signaling, thereby driving macrophages toward glycolytic metabolism. As a consequence, macrophages not only increase their pro-inflammatory output but also lose their capacity for tissue remodeling. In parallel, TKIs and mTOR inhibitors may disturb immunometabolic thresholds (43), indirectly impairing macrophage migration and phagocytic function.

When these targeted therapies are combined clinically with glucocorticoids or chemotherapeutic agents, the pathological synergy between local inflammation and defective repair is further intensified. In addition to inducing systemic immunosuppression, such regimens compromise the mucosal barrier and increase the risk of recurrent infection (1, 42, 44).

Within this extreme multidrug-conditioned microenvironment, macrophages are simultaneously exposed to several layers of pathological stress: denosumab-induced arrest of bone remodeling and loss of reparative cues, antiangiogenic therapy-driven hypoxia and pro-inflammatory pressure, and systemic treatment-associated immunometabolic disturbance. Taken together, as summarized in **Table 1**, these agents converge on a shared pathogenic framework for MRONJ by altering the metabolic and microenvironmental constraints that shape macrophage behavior, thereby systematically reducing the likelihood of successful inflammation resolution and progression toward tissue repair (45).

**Table 1:** Drug class-specific mechanisms of macrophage dysfunction in MRONJ.

Drug class	Primary macrophage-relevant mechanism	Functional consequence	References
Nitrogen-containing bisphosphonates	Mevalonate pathway blockade and defective small GTPase prenylation	Impaired efferocytosis and persistence of a pro-inflammatory state	(19, 20, 31)
RANKL inhibitors	Disruption of osteoimmune remodeling cues	Delayed transition from inflammation to repair	(37, 38)
Antiangiogenic agents/TKIs	Hypoxia-driven immunometabolic stress	Reduced migratory/phagocytic capacity and impaired repair support	(12, 46, 47)
Glucocorticoids/chemotherapy	Systemic immune suppression and mucosal barrier injury	Defective immune recruitment, host defense, and coordinated healing	(1, 42, 44)

## 4 A macrophage-centered immunopathological network: functional module mismatch and failed reparative coupling

The immunopathology of MRONJ is not adequately captured by a simple binary opposition between “excessive inflammation” and “immunosuppression.” Rather, it represents a network of functional module mismatch centered on macrophages. Under the combined influence of drug-induced metabolic perturbation and local microenvironmental stress, the macrophage-governed clearance–resolution–repair program becomes progressively disrupted, rendering the pathological process difficult to reverse.

### 4.1 Polarization and functional state: from the M1/M2 paradigm to disordered wound-repair programming

Wound healing in the oral mucosa and jawbone generally follows a predictable temporal sequence: the early phase is dominated by pathogen clearance and inflammatory control, whereas the intermediate and late phases progressively shift toward matrix remodeling, angiogenesis, and epithelial closure. Macrophages are involved throughout this process, and their phenotypes are not fixed in static M1 or M2 states, but instead represent dynamic functional programs shaped by the surrounding microenvironment (45).

However, multiple clinical biopsy studies and animal models have shown that macrophages within MRONJ lesions exhibit a pathological state of functional arrest or hybrid activation. On the one hand, persistent stimulation from necrotic bone and bacterial products sustains the high expression of M1-associated markers such as iNOS, TNF-α, and IL-6 (48). On the other hand, drug-induced metabolic blockade prevents an effective transition from the inflammatory state toward a reparative phenotype, resulting in insufficient expression or functional impairment of repair-associated mediators such as CD206 and IL-10. This paradoxical state is essentially a functional deadlock: macrophages are locked in an activated state by persistent damage-associated molecular patterns (DAMPs) and pathogen-associated molecular patterns (PAMPs), yet are unable to eliminate the offending stimuli because of cytoskeletal paralysis. At the same time, they fail to initiate downstream tissue repair programs, ultimately leading to non-healing wounds (29, 48).

### 4.2 A key mechanistic axis: defective efferocytosis and failure of inflammation resolution

Efferocytosis, the process by which macrophages engulf and clear apoptotic cells such as senescent neutrophils, is a prerequisite for the successful resolution of inflammation (49). Under physiological healing conditions, timely efferocytosis prevents apoptotic cells from undergoing membrane rupture and secondary necrosis. In MRONJ, however, this clearance mechanism is profoundly disrupted, and uncleared apoptotic cells may progress to secondary necrosis (50), initiating a pathogenic cascade that converges with excessive activation of the NLRP3 inflammasome.

Impaired efferocytosis in MRONJ may involve abnormalities at both the recognition and engulfment stages. At the level of recognition, increased protease activity within the inflammatory milieu may compromise the function of efferocytosis receptors such as MerTK, as suggested by related inflammatory models, thereby weakening the ability of macrophages to sense “eat-me” signals (51). At the execution stage, zoledronic acid-induced impairment of Rac1 prenylation has been shown to disrupt actin remodeling and phagocytic cup formation in experimental BRONJ models (28). As a consequence, apoptotic neutrophils and osteoblasts that fail to be cleared undergo secondary necrosis, and the released damage-associated molecular patterns (DAMPs) continuously activate Toll-like receptors and inflammasome pathways, establishing a self-amplifying necrosis–inflammation loop (11, 52).

Secondary necrotic products may provide activating signals for NLRP3 inflammasome assembly and activation. Additional contributors to this “signal 2” include hydroxyapatite microcrystals on exposed necrotic bone surfaces, locally elevated extracellular ATP, and lysosomal membrane damage caused by frustrated phagocytosis (30). In parallel, bacterial biofilms within the oral microenvironment, particularly lipopolysaccharide (LPS), act in concert with drug-related stress to sustain NF-κB-dependent priming in macrophages (signal 1) (7, 29, 53).

Under the convergence of these two signals, persistent and aberrant NLRP3 inflammasome activation may occur in macrophages. This complex not only drives extensive processing and release of the major pro-inflammatory cytokines IL-1β and IL-18, but may also trigger macrophage pyroptosis, thereby further amplifying local suppurative inflammation (54). These inflammatory effects may further disturb osteoimmune coupling, osteogenic activity, and angiogenic repair, although the direct contribution of this pathway in human MRONJ remains incompletely defined (38).

Collectively, the available evidence supports a model in which impaired efferocytosis, accumulation of damage-associated signals, and inflammasome activation may reinforce one another and contribute to persistent inflammation and disrupted tissue repair in MRONJ.

A relatively underexplored component of this network is osteocyte–macrophage crosstalk. Under drug exposure, inflammatory stress, and mechanical loading, osteocyte apoptosis or necrosis may release damage-associated signals and alter RANKL/OPG- and VEGF-associated remodeling cues (55), thereby influencing macrophage and osteoclast-lineage responses (56). Conversely, osteal macrophages may contribute to the clearance of dying bone-lineage cells and support reparative coupling (17, 24, 57). Disruption of this bidirectional communication may sustain innate immune activation and impair coordinated bone remodeling in MRONJ. However, direct evidence defining osteocyte–macrophage crosstalk in human MRONJ remains limited; this axis should therefore be viewed as a plausible and testable component of the broader osteoimmune network rather than an established causal pathway.

### 4.3 Crosstalk with adaptive immunity: interaction and imbalance of the Th17/Treg axis

The functional state of macrophages exerts a direct influence on adaptive immune responses. Within the chronic inflammatory microenvironment of MRONJ, macrophage-derived IL-6, IL-23, and IL-1β collectively drive the differentiation of CD4+ T cells toward the Th17 lineage while simultaneously suppressing the differentiation and expansion of regulatory T cells (Tregs) (58).

Th17 cells produce IL-17, a potent pro-inflammatory cytokine that recruits neutrophils and promotes osteoclastogenesis to facilitate the removal of necrotic bone. In the context of MRONJ, however, antiresorptive agents have already systemically suppressed osteoclast activity. As a result, IL-17-driven osteoclastogenic signals fail to generate effective bone resorption for sequestrum separation; instead, they further exacerbate local neutrophil infiltration and immune-mediated tissue damage (45). In contrast to excessive Th17 activation, both the number and function of Tregs, which are essential for immunosuppression and tissue repair, are markedly impaired. Notably, restoration of the Treg/Th17 balance by mesenchymal stem cell infusion has been shown to reverse MRONJ-like lesions, underscoring that correction of this adaptive immune imbalance is a critical step toward re-establishing local homeostasis (59).

### 4.4 Biofilm– vascular–macrophage interplay: closure of a self-perpetuating pathogenic loop

The refractory nature of MRONJ may arise from a self-reinforcing pathological loop in which persistent biofilm stimulation, neutrophil/NET-associated vascular injury, and macrophage clearance failure amplify one another within the local microenvironment.

Exposed bone surfaces in MRONJ are frequently covered by complex polymicrobial biofilms composed of organisms such as Actinomyces and Fusobacterium (16). By secreting an extracellular polysaccharide matrix, these microbial communities form a dense physical barrier (7) that impedes effective antibiotic penetration while serving as a persistent source of pathogen-associated molecular patterns (PAMPs), including lipopolysaccharide (LPS). As a result, the local tissue remains in a state of sustained immune activation.

Persistent bacterial stimulation recruits large numbers of neutrophils to the lesion, where they counter infection through NETosis, releasing neutrophil extracellular traps (NETs) composed of a DNA scaffold, histones, and antimicrobial enzymes (60). Although NETs provide antimicrobial defense and structural containment, they also exert substantial cytotoxic effects on surrounding host tissues (60, 61). Excessive or insufficiently cleared NETs may injure endothelial cells and promote microvascular thrombosis, thereby impairing local perfusion (60). Although not directly demonstrated in human MRONJ, this process could exacerbate hypoxia and acidosis and further compromise macrophage cytoskeletal function and efferocytosis. Under physiological conditions, macrophages contribute to the timely removal of these immune remnants through DNase production and efferocytosis (62). In the pathological microenvironment of MRONJ, however, macrophages are likely to exhibit inadequate NET-remnant clearance together with defective phagocytic capacity, potentially contributing to pathological NET accumulation at the lesion site.

Retained NETs may become a persistent source of pro-inflammatory signals and further compromise macrophage-mediated clearance (62). Neutrophil recruitment and NET accumulation may therefore connect persistent biofilm stimulation with vascular dysfunction. This biofilm–vascular–macrophage loop may contribute to the failure of both soft- and hard-tissue healing in MRONJ (**Figure 2**).

**Figure 2:**
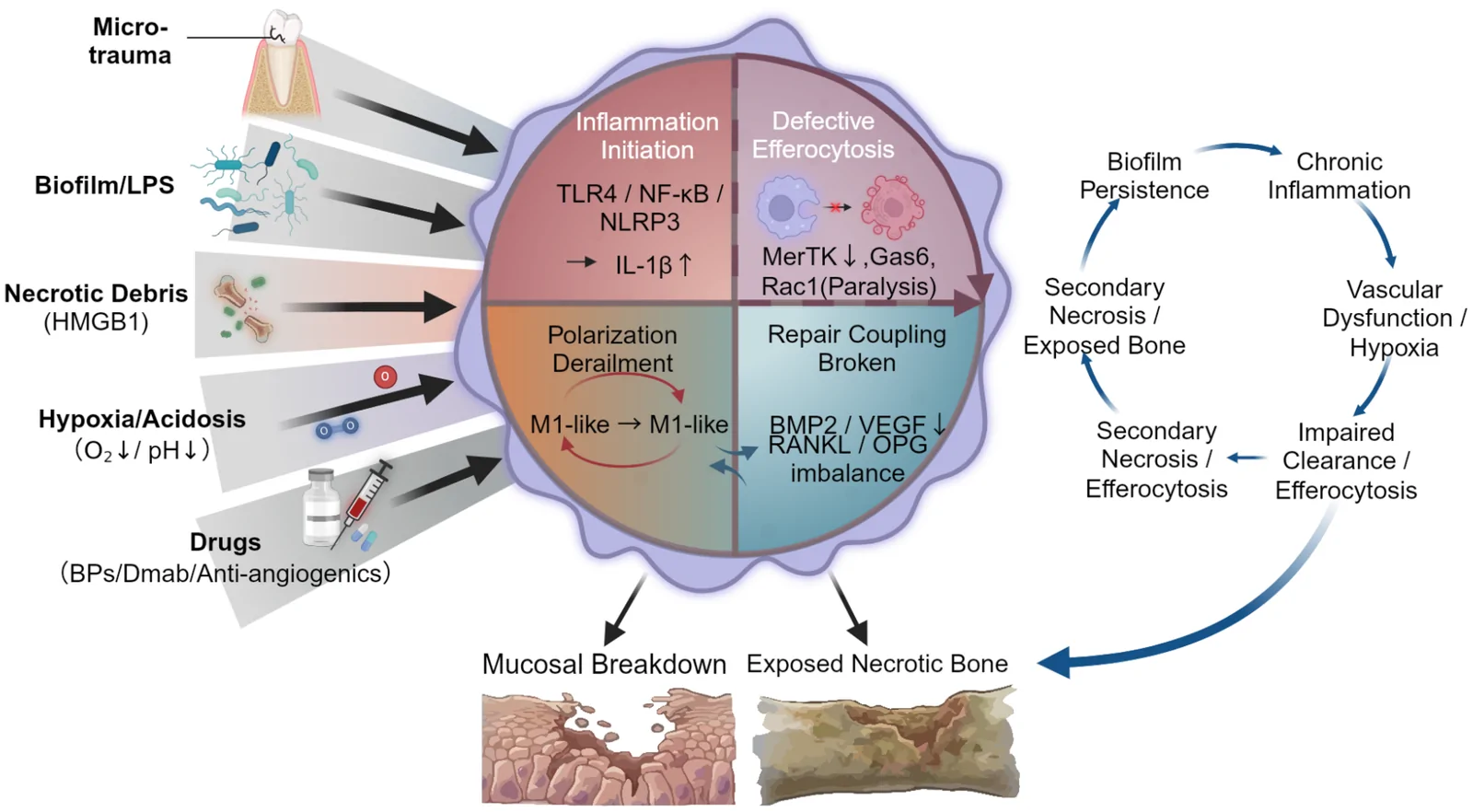
Proposed macrophage-centered network of impaired inflammation resolution in MRONJ. Drug-related stress, extraction injury, and biofilm persistence may promote pro-inflammatory macrophage reprogramming and defective efferocytosis (7, 28–30, 51, 53). Neutrophil/NET-associated vascular dysfunction may aggravate hypoxia and impair macrophage clearance (60). Disrupted osteoimmune coupling and adaptive immune imbalance may further compromise tissue repair (37, 46, 58, 59). These interactions may form a self-reinforcing biofilm–vascular–macrophage loop and contribute to mucosal breakdown and persistent exposure of necrotic bone. Created in BioRender. Quad, W. (2026) https://BioRender.com/ohqqivr.

## 5 Evidence overview: from basic research to clinical observations

This section aims to integrate evidence across multiple levels in order to construct a coherent framework for the immunopathological mechanisms underlying MRONJ. However, the available evidence remains heterogeneous in both design and strength, ranging from descriptive clinical pathology to mechanistic studies in animal and *in vitro* models. Using clinical histopathology as the *in situ* pathological baseline, we further examine disease progression along two complementary dimensions, namely, temporal dynamics and functional intervention, to clarify the mechanisms driving MRONJ initiation and progression.

### 5.1 Clinical histopathology: macrophage phenotypic heterogeneity and pathological hybrid states

Clinical immunohistochemical studies have shown that MRONJ lesions are not only enriched in CD68+ macrophages, but also exhibit marked phenotypic heterogeneity. Unlike conventional osteomyelitis, macrophages in MRONJ do not simply polarize toward a canonical M1 or M2 phenotype under the combined stresses of infection and hypoxia. Instead, they assume a hybrid pathological state characterized by persistent expression of pro-inflammatory markers, such as iNOS, combined with impaired reparative function (48). This pattern reflects a lesion microenvironment in which inflammation remains active but resolution is blocked. These macrophage populations, which accumulate around necrotic bone yet fail to effectively clear it, constitute the histopathological foundation of the immune-mediated failure of healing in MRONJ.

### 5.2 Temporal dynamics in animal models: disruption of the sequence of inflammation resolution and repair

Whereas clinical specimens provide a static snapshot of MRONJ, animal models reveal the dynamic trajectory of disease progression (20). During normal extraction socket healing, the immune microenvironment undergoes an orderly temporal transition from inflammatory clearance to tissue reconstruction. In MRONJ, however, this sequence is profoundly disrupted (**Figure 3**). A recent animal study showed that the jaw-specific combination of high microbial burden and barrier vulnerability forces macrophages to remain in a prolonged pro-inflammatory phase, thereby impairing the transition from M1-like to reparative states (20, 23). As a consequence, although inflammatory initiation may proceed normally, the resolution phase fails to occur effectively. This leads to persistent retention of uncleared necrotic bone fragments and empty osteocytic lacunae, ultimately resulting in failure of soft tissue closure (45).

**Figure 3:**
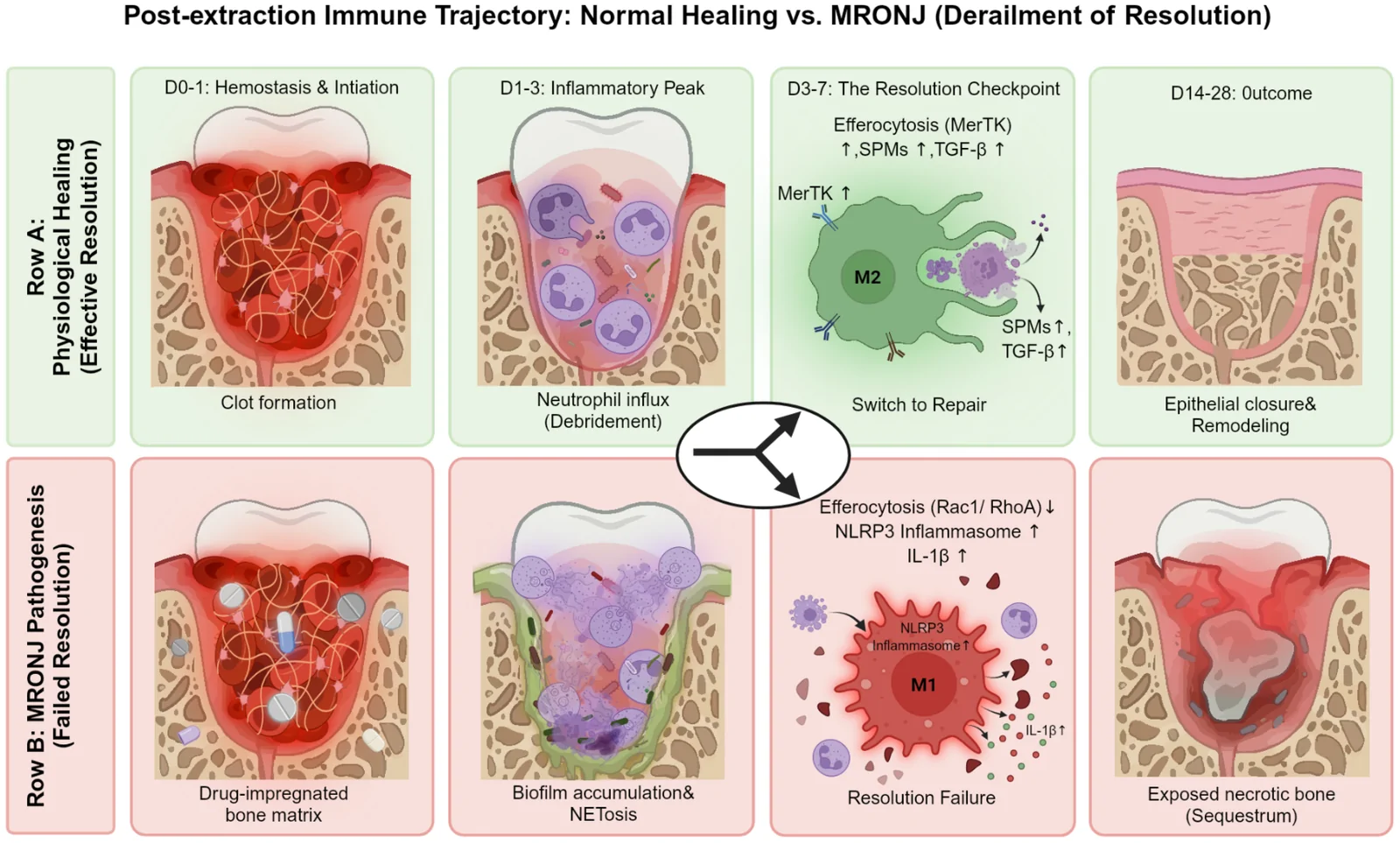
Proposed post-extraction immune trajectories in physiological healing and MRONJ. **(A)** Physiological healing progresses from early inflammation to resolution and repair, aided by efferocytosis and MerTK-mediated clearance (10, 11, 23, 50, 51). **(B)** In MRONJ, drug exposure, persistent inflammatory macrophages, and biofilms may prevent this transition (20, 23, 29, 53). Defective Rac1/RhoA-dependent efferocytosis and sustained NLRP3 signaling may delay repair and contribute to bone exposure and sequestration (28, 30). The time windows are schematic and may differ among models and clinical settings. Created in BioRender. Quad, W. (2026) https://BioRender.com/q21y63n.

### 5.3 Functional intervention studies: probing the functional contribution of macrophage dysfunction

Functional intervention studies provide stronger mechanistic evidence than observational analyses and suggest that the integrity of the macrophage clearance–resolution program may be an important determinant of MRONJ-associated healing failure.

“Molecular rescue” experiments targeting the mevalonate pathway have shown that exogenous supplementation with geranylgeraniol (GGOH) can bypass bisphosphonate-induced blockade, restore Rac1/RhoA prenylation, and reverse macrophage cytoskeletal paralysis and efferocytic defects. Once phagocytic function is restored, clearance of necrotic bone and soft tissue healing are significantly improved, supporting the possibility that macrophage dysfunction may contribute to early disease development rather than merely arise as a consequence of established tissue necrosis (28, 63).

Cell-based immunotherapeutic studies provide complementary support for the feasibility of restoring local immune homeostasis. When endogenous macrophage function is impaired, infusion of mesenchymal stem cells (MSCs) or administration of MSC-derived exosomes can effectively suppress local Th17 responses and re-establish Treg-mediated immune balance (59, 64). Together, these findings indicate that interventions that restore immune balance and macrophage-associated reparative functions can improve healing outcomes in experimental MRONJ-like models.

### 5.4 Evidence integration and resolution of current controversies

Taken together, the available evidence (**Table 2**) supports the immune landscape framework proposed by Zhang et al. (15) and is broadly compatible with the macrophage-centered framework proposed in this review: MRONJ represents a state of dysregulated innate and adaptive immunity within a permissive local microenvironment.

**Table 2:** Key evidence supporting a macrophage-centered mechanism in MRONJ.

Evidence type	Experimental setting	Main finding	References
Clinical pathology	Human MRONJ biopsy specimens	Enrichment of pro-inflammatory macrophages and reduced reparative phenotypes within lesions	(48)
Temporal dynamics	Zoledronic acid-associated murine extraction models	Prolonged inflammatory activation and impaired transition toward repair during socket healing	(23)
Functional perturbation	Systemic macrophage depletion	Aggravated necrotic lesion progression and defective wound debridement	(65)
Mechanistic rescue	GGOH supplementation *in vitro*/*in vivo*	Restoration of prenylation-dependent macrophage function and efferocytosis	(28, 63)
Immune restoration	MSCs, exosomes, or pro-resolving interventions	Improved immune balance and healing-associated tissue repair	(59, 66, 67)

Several ongoing controversies in the field, including whether infection acts primarily as an initiating event or a sustaining factor (1, 53), and how immunosuppression can coexist with excessive inflammation (45, 48), can be reconciled within the conceptual framework of failed inflammation resolution. This framework suggests that it is the impairment of macrophage functional programs that allows infection to persist, thereby explaining the paradoxical coexistence of elevated pro-inflammatory cytokine expression and arrested tissue repair. Accordingly, restoration of endogenous resolution programs, rather than simple suppression of inflammation or eradication of microbes alone, may represent a more promising therapeutic avenue for future MRONJ management (48).

## 6 Therapeutic opportunities: translational strategies targeting macrophages

From a translational perspective, macrophage-targeted therapy for MRONJ should not be limited to the simple suppression of inflammation, but should instead aim to reconstruct the immune–repair cascade following tooth extraction or minor tissue injury. The central therapeutic goal is to limit early inflammatory amplification while preserving antimicrobial defense, restore the clearance of necrotic bone and apoptotic cells through efferocytosis, promote inflammation resolution, and ultimately re-establish coupling with angiogenesis and tissue remodeling. On this basis, the potential interventional approaches discussed in this section can be broadly categorized into four major dimensions (**Table 3**).

**Table 3:** Macrophage-targeted therapeutic strategies for MRONJ.

Strategy	Targeted module	Potential benefit	Evidence level	References
Local antimicrobial/antiseptic measures	Biofilm burden and persistent inflammatory stimulation	Indirect support for macrophage-mediated healing by reducing local immune stress	Clinical (guideline/observational)	(1)
NLRP3 or IL-1 pathway inhibition	Inflammasome activation and inflammatory amplification	Limitation of excessive cytokine release and secondary tissue damage	Preclinical (*in vitro*/animal)	(88, 89)
Mevalonate pathway rescue (GGOH)	Prenylation-dependent cytoskeletal remodeling and efferocytosis	Restoration of macrophage clearance function	Preclinical (*in vitro*/MRONJ animal)	(28, 32)
Specialized pro-resolving mediators (e.g., RvD1/RvE1)	Efferocytosis and inflammation resolution	Promotion of debris clearance and resolution-associated repair	Preclinical (*in vitro*/animal; indirect)	(67, 90, 91)
MSC-derived exosomes or immunomodulatory biomaterials	Local immune–repair recoupling	Improvement of angiogenesis, tissue sealing, and pro-repair microenvironment remodeling	Preclinical (*in vitro*/animal; partly MRONJ-specific)	(64, 66, 81–83)

MRONJ, medication-related osteonecrosis of the jaw; GGOH, geranylgeraniol; SPMs, specialized pro-resolving mediators; MSC, mesenchymal stem cell.

Clinical evidence is indirect for macrophage-related outcomes, and no macrophage-targeted intervention has yet been validated in clinical trials for MRONJ.

### 6.1 Clinical risk management: a first-line strategy for lowering the inflammatory threshold

Perioperative risk management remains the most immediately feasible strategy in current clinical practice. Risk stratification based on a comprehensive assessment of medication history, comorbidities, and oral infectious status (1, 68), together with optimization of surgical timing and reinforcement of intraoperative infection control and primary soft tissue closure, can substantially reduce the persistent microbial and damage-related stimuli associated with wound exposure (1). A large body of clinical observational evidence indicates that the local infectious burden and the quality of mucosal closure are major determinants of MRONJ outcomes (15, 69).

It should be noted, however, that the supporting evidence for these measures is largely correlative. The effectiveness of a “drug holiday” varies across different drug classes and patient populations, and in patients with malignancy, such decisions must also be balanced against the risk of skeletal-related events (1). Therefore, these approaches should be regarded primarily as foundational strategies for lowering the baseline level of local inflammatory stress, rather than as causal interventions targeting specific immune pathways.

### 6.2 Modulating macrophage inflammatory states: metabolic and signaling reprogramming

Interventions targeting macrophages should not remain confined to a static M1/M2 framework, but should instead focus on key functional readouts, including phagocytosis, efferocytosis, pro-angiogenic activity, and tissue sealing (70, 71).

As an important node in immunometabolic regulation, PPARγ can attenuate selected pro-inflammatory programs by reshaping transcriptional and metabolic networks, while promoting macrophage functional states that are more compatible with tissue repair (72, 73). However, systemic administration of PPARγ agonists may cause metabolic adverse effects, and in the microbe-rich oral environment, pro-repair activity must be carefully balanced against the preservation of antimicrobial defense. For this reason, short-course local delivery may represent a more appropriate strategy.

In addition, inhibition of NF-κB signaling and the NLRP3 inflammasome may suppress the release of IL-1β and IL-18, thereby limiting inflammatory amplification (74, 75). Nevertheless, a mere reduction in inflammatory intensity is not equivalent to restoration of the resolution program (71). Moreover, in the high-burden oral microbial environment, systemic anti-inflammatory therapy may increase the risk of opportunistic oral infections, underscoring the need for precise control of the therapeutic window for local application.

### 6.3 Restoring efferocytosis and promoting resolution: a pivotal axis for reversing pathology

If the immunopathological core of MRONJ is understood as insufficient clearance of necrotic and apoptotic cells combined with impaired resolution of inflammation, then enhancing efferocytosis and activating pro-resolving programs may offer a more specific and physiologically grounded strategy than simple anti-inflammatory suppression (50, 71, 76).

Specialized pro-resolving mediators (SPMs), such as resolvin D1, can promote macrophage phagocytosis and efferocytosis through receptors including FPR2/ALX, while limiting excessive neutrophil infiltration and driving the orderly transition from inflammation to repair (14, 77). Unlike conventional anti-inflammatory drugs, SPMs are distinguished by their ability to promote resolution without broadly suppressing host defense (78), which, in theory, makes them particularly well suited to maintaining the balance between host defense and tissue repair in the microbe-rich oral environment.

At present, most supporting evidence derives from animal models and from studies of other inflammatory or wound-healing conditions, whereas direct evidence in MRONJ remains limited. Therefore, a more realistic translational strategy may be to apply these agents as a short-course, localized adjunctive therapy following tooth extraction or surgical debridement.

### 6.4 Cells, exosomes, and biomaterials: localized delivery tailored to the microenvironment

Given the localized nature of MRONJ and the moist, microbe-rich environment of the oral cavity, local delivery platforms offer distinct therapeutic advantages (79).

Mesenchymal stem cell (MSC)-derived exosomes have opened a new paradigm for cell-free therapy (80). Enriched in miRNAs and anti-inflammatory proteins, these vesicles may modulate local inflammatory and reparative signaling, thereby reshaping the immune–repair microenvironment (64, 66). At the same time, functionalized biomaterials provide dual benefits as both physical scaffolds and immunomodulatory platforms (81, 82). Injectable immunomodulatory hydrogels can fill irregular extraction sockets and provide sustained local delivery of bioactive molecules, thereby creating a microenvironment favorable for reparative macrophage programming (83). Clinically used platelet-rich fibrin (PRF) may further accelerate healing by forming an autologous immune-supportive barrier (84). In addition, physical modalities such as photobiomodulation (PBM) have also been shown to promote repair-associated macrophage functions by modulating mitochondrial reactive oxygen species (ROS) levels (85, 86).

Future translational challenges include standardizing quality control for exosome-based products and achieving precise regulation of biomaterial degradation kinetics (87). At present, the most realistic path toward clinical translation is to use these biomaterial-based strategies as adjuncts to standard surgical debridement rather than as stand-alone therapies (1).

## 7 Perspectives and limitations: toward a resolution-centered paradigm for MRONJ management

Although a macrophage-centered framework provides a useful unifying perspective on MRONJ pathogenesis (15), a substantial gap remains between mechanistic insight and clinical translation (92). Key unresolved questions include which macrophage subsets are most susceptible to MRONJ-associated medications, at what stage the macrophage clearance–resolution program becomes persistently impaired, and whether these alterations represent a shared pathogenic mechanism across different drug classes. Addressing these questions will require longitudinal, spatially resolved, and functionally oriented studies that integrate high-resolution spatiotemporal mapping with functional omics approaches (93, 94). Such efforts may ultimately support the identification of clinically accessible biomarkers and the development of stage-specific interventions guided by mechanisms of inflammation resolution (1, 14).

### 7.1 From single markers to spatial atlases: mapping the jaw-specific immune microenvironment

Most current studies rely on flow cytometry or conventional histological sections and therefore lack decisive *in situ* spatial information (93). A major future breakthrough will depend on integrating spatial transcriptomics with single-cell RNA sequencing (scRNA-seq) to generate a jaw-specific spatiotemporal immune atlas (94). Such an approach would enable a more testable set of core questions to be addressed: whether distinct macrophage subsets with unique susceptibilities to drug exposure exist within the niche at the bone–soft tissue interface (10), and how disrupted or “silent” intercellular communication between macrophages and adjacent nerves, vasculature, and epithelium impedes the reparative process (95), thereby blocking the transition from inflammation to tissue repair. Deciphering these spatiotemporal interaction networks may ultimately translate the concept of jaw-specific susceptibility into identifiable molecular switches and actionable therapeutic windows.

### 7.2 Functional priorities: from surface phenotypes to efferocytosis, resolution, and immunometabolic profiling

Given that the conventional M1/M2 dichotomy is insufficient to resolve the complexity of a microenvironment shaped simultaneously by necrosis and infection (96), evaluation frameworks should shift from a primary focus on cellular phenotype toward one centered on cellular function (97). In this context, we propose an integrated assessment system built around three core dimensions: efferocytic capacity, the profile of specialized pro-resolving mediators (SPMs), and immunometabolic flux (76). For example, the phagocytic index measured using fluorescently labeled apoptotic cells may provide a direct readout of efferocytosis efficiency (13), while lipidomic profiling of the balance between resolvins and pro-inflammatory lipid mediators can more accurately reflect the true reparative potential of a lesion than static immunohistochemical markers. This framework would define MRONJ more precisely as a form of functional immune disequilibrium characterized by impaired clearance–resolution programming, thereby overcoming the limitations of assessments based solely on inflammatory intensity (92).

### 7.3 Translational priorities: biomarkers, patient stratification, and resolution-centered clinical trials

Clinical translation should focus on two major dimensions. First, with respect to biomarker development, there is an urgent need to translate emerging transcriptomic and molecular signatures of MRONJ into accessible biomarkers detectable in saliva, gingival crevicular fluid, or blood, thereby enabling earlier risk prediction and improved patient stratification (98). Compared with conventional imaging-based approaches, such markers may offer a more sensitive means of identifying high-risk patients before overt clinical progression.

Second, in the design of clinical trial endpoints, future interventional studies should not rely solely on symptomatic outcomes such as pain relief or reduction in exudation (92). Instead, key endpoints should include the rate of complete mucosal closure, the time to sequestrum separation, and the restoration of the local immune microenvironment, as assessed by parameters such as the efferocytosis index, pro-resolving mediator profiles, and bacterial burden or biofilm-related readouts (1, 14).

From a therapeutic perspective, macrophage-targeted approaches are likely to complement, rather than replace, established surgical and infection-control strategies (1, 99). Such interventions may need to be tailored to disease stage and treatment timing (29). In high-risk patients undergoing dentoalveolar surgery, perioperative preventive strategies could focus on preserving macrophage clearance functions and promoting mucosal healing (1, 66). By contrast, established lesions may require an integrated approach combining surgical debridement and microbial control with localized pro-resolving or immunomodulatory therapies. In this context, local delivery of specialized pro-resolving mediators, metabolic rescue agents, or immunomodulatory biomaterials represents a rational avenue for further preclinical evaluation and subsequent clinical investigation (28, 66, 100).

Taken together, a clinical evaluation framework centered on inflammation resolution, durable mucosal closure, and tissue remodeling may enhance the ability of future trials to detect clinically meaningful treatment effects and facilitate a shift in MRONJ management from symptom control toward restoration of coordinated tissue repair (78).

### 7.4 Limitations of the current evidence and the proposed framework

Although the macrophage-centered framework provides a coherent perspective on MRONJ immunopathology, several limitations should be acknowledged. The current evidence base remains heterogeneous and is derived largely from cross-sectional histopathological analyses of human specimens, animal models, and *in vitro* mechanistic studies. Because human biopsy specimens are generally obtained from established lesions (101), they cannot reliably define temporal sequence or establish causality. It therefore remains uncertain whether local macrophage dysfunction contributes to disease initiation or develops secondarily in response to osteonecrosis, microbial colonization, tissue hypoxia, and mucosal breakdown.

Several mechanistic links discussed in this review—including defective efferocytosis, impaired MerTK-dependent recognition, altered FPR2–RhoA signaling and Rac1-dependent cytoskeletal remodeling (28), persistent NLRP3 inflammasome activation (30, 66), and osteocyte–macrophage crosstalk—have not been established as components of a unified causal pathway in human MRONJ. Some of these mechanisms remain partly extrapolated from related inflammatory, skeletal, or wound-healing models. The strength of mechanistic evidence also varies across drug classes. Relatively direct experimental evidence links nitrogen-containing bisphosphonates to impaired protein prenylation and macrophage cytoskeletal dysfunction, whereas direct evidence for the macrophage-specific effects of denosumab, antiangiogenic agents, and multidrug regimens remains limited (102).

Finally, MRONJ is a multifactorial disorder involving interactions among immune and skeletal cells, vascular and mucosal tissues, mechanical injury, and the oral microbiota (103). Macrophage dysregulation should therefore be viewed as an interconnected component of a broader osteoimmune network rather than as a single or uniformly dominant pathogenic driver (45). Accordingly, the proposed framework should be regarded as an integrative and testable model that requires further validation in longitudinal human studies and through spatially resolved, single-cell, and cell-specific functional approaches.

## 8 Conclusion

In summary, MRONJ is not merely an isolated bone disorder, but may also be viewed as a form of functional immune disequilibrium arising within a specialized osteoimmune niche. Its central pathological axis may be conceptualized as a sequence linking drug-induced metabolic perturbation, macrophage efferocytic dysfunction, and failure of inflammation resolution. This disruption of tissue homeostasis, driven by efferocytic failure, profoundly compromises the clearance of necrotic debris and dying cells, thereby converting what should be a self-limited inflammatory response into sustained tissue-destructive inflammation.

Accordingly, future MRONJ treatment strategies may increasingly extend beyond the removal of necrotic bone to include adjunctive approaches aimed at restoring local immune and reparative homeostasis. Therapeutic strategies aimed at reactivating macrophage efferocytosis, combined with biomaterial-based remodeling of the local microenvironment toward a pro-reparative state, warrant further evaluation as adjunctive approaches for improving tissue repair while preserving infection control. This conceptual transition, moving from a bone necrosis-centered model to a resolution-centered paradigm, not only deepens our mechanistic understanding of MRONJ, but also provides a strong theoretical foundation for the development of novel interventions aimed at restoring tissue homeostasis as the ultimate therapeutic endpoint.
